# Osteogenic Oxysterol, 20(*S*)-Hydroxycholesterol, Induces Notch Target Gene Expression in Bone Marrow Stromal Cells

**DOI:** 10.1359/jbmr.091024

**Published:** 2009-10-17

**Authors:** Woo-Kyun Kim, Vicente Meliton, Sotirios Tetradis, Gerry Weinmaster, Theodore J Hahn, Marc Carlson, Stanley F Nelson, Farhad Parhami

**Affiliations:** 1 Department of Medicine, UCLA School of MedicineLos Angeles, CA, USA; 2 UCLA School of DentistryLos Angeles, CA, USA; 3 Department of Biological Chemistry, UCLA School of MedicineLos Angeles, CA, USA; 4 VA Greater Los Angeles Healthcare System and Geriatric Research, Education, and Clinical CenterLos Angeles, CA, USA; 5 Department of Human Genetics, UCLA School of MedicineLos Angeles, CA, USA

**Keywords:** oxysterol, mesenchymal stem cells, Notch, hedgehog, osteogenesis

## Abstract

We previously reported that specific oxysterols stimulate osteogenic differentiation of pluripotent bone marrow stromal cells (MSCs) through activation of hedgehog (Hh) signaling and may serve as potential future therapies for intervention in osteopenia and osteoporosis. In this study we report that the osteogenic oxysterol 20(*S*)-hydroxycholesterol (20*S*) induces the expression of genes associated with Notch signaling. Using M2-10B4 (M2) MSCs, we found that 20*S* significantly induced *HES-1*, *HEY-1*, and *HEY-2* mRNA expression compared with untreated cells, with maximal induction after 48 hours, whereas the nonosteogenic oxysterols did not. Similar observations were made when M2 cells were treated with sonic hedgehog (Shh), and the specific Hh pathway inhibitor cyclopamine blocked 20*S*-induced Notch target gene expression. 20*S* did not induce Notch target genes in *Smo*^−/−^ mouse embryonic fibroblasts, further confirming the role of Hh signaling in 20*S*-induced expression of Notch target genes. Despite the inability of liver X-receptor (LXR) synthetic ligand TO901317 to induce Notch target genes in M2 cells, LXR knockdown studies using siRNA showed inhibition of 20*S*-induced *HEY-1* but not *HES-1* expression, suggesting the partial role of LXR signaling in MSC responses to 20*S*. Moreover, 20*S*-induced Notch target gene expression was independent of canonical Notch signaling because neither 20*S* nor Shh induced CBF1 luciferase reporter activity or NICD protein accumulation in the nucleus, which are hallmarks of canonical Notch signaling activation. Finally, *HES-1* and *HEY-1* siRNA transfection significantly inhibited 20*S*-induced osteogenic genes, suggesting that the pro-osteogenic effects of 20*S* are regulated in part by *HES-1* and *HEY-1*. © 2010 American Society for Bone and Mineral Research

## Introduction

The Notch signaling pathway is an evolutionarily conserved intercellular signaling mechanism that plays a prominent role in cell proliferation, differentiation, and survival.(1,2) The canonical Notch signaling pathway is activated when Notch receptors (Notch-1, -2, -3, and -4) interact with ligands [Jagged-1 and -2 and Delta-like (Dll-1, -3, and -4)] on adjacent cells, triggering proteolytic cleavage of the receptor by the presenilin–γ-secretase complex.(1,2) This releases the Notch intracellular domain (NICD), which translocates to the nucleus and binds the CBF-1 DNA-binding protein, thereby inducing the expression of Notch target genes, including the isoforms of *HES* (*HES-1*, *-3*, and *-5*) and *HEY* (*HEY-1*, *-2*, and *-3*).(3) These Notch target genes are involved in various biologic processes, including angiogenesis, osteogenesis, adipogenesis, myogenesis, somatogenesis, and neurogenesis.(4–9) Regulation of Notch signaling pathway and target gene expression is important in embryonic and postembryonic development and tissue homeostasis.(1,10–12) However, it remains controversial as to whether Notch signaling acts as a positive or negative regulator of osteogenic differentiation in osteoblast progenitor cells and bone formation. For example, Dll-3- or presenilin-1-deficient mice exhibit severe skeletal defects,(8,13,14) and overexpression of Notch-1, HES-1, or HEY-1 enhances osteogenic differentiation of MSCs(15–17) in part through positive regulation of and cooperation with Runx2, suggesting that Notch signaling may play positive roles in bone formation. On the other hand, *presenilin-2* null mice have greatly increased trabecular bone mass, and HES or HEY proteins were shown to inhibit Runx2 transcriptional activity in CHO and ST2 cells, suggesting the negative role of Notch signaling in osteogenesis.(18) However, it also has been suggested that *HES* and/or *HEY* expression induced by Notch signaling may be important in regulating bone density during aging by maintaining a sufficient pool of bone marrow progenitor cells for osteogenesis.(18) Therefore, further examination of the role of Notch signaling in regulating osteogenesis and bone formation is required, and it is likely that the differences in the reports cited earlier may be due to differences in the specific experimental models used in studying the role of Notch signaling in osteogenesis.

In addition to canonical Notch signaling, the expression of Notch target genes is regulated by growth factors, including transforming drowth factor β (TGF-β), bone morphogenetic protein (BMP), vascular endothelial growth factor (VEGF), and sonic hedgehog (Shh).(17,19–21) TGF-β induces HEY-1 and Jagged-1 in epithelial cells from mammary gland, kidney tubules, and epidermis,(19) and BMP-9 induces HEY-1 expression in C3H10T1/2 cells.(17) Also, Shh and VEGF induce *Notch-5* and *HES-1* mRNA expression in various cells, including C3H10T1/2 cells, MNS70 neural cells, and granule neuron precursors.(20–22) Moreover, it has been suggested that regulation of HES-1 expression by c-Jun kinase signaling and Hedgehog signaling may be mediated through the activation of noncanonical Notch signaling pathways.(22–24) Hence the molecular mechanisms by which growth and differentiation factors activate the Notch signaling pathway and induce the expression of Notch target genes require further elucidation.

Oxysterols, a large family of 27-carbon oxygenated products of cholesterol present in the circulation and in human and animal tissues,(25) are involved in various biologic and pathologic processes, including cholesterol efflux, lipoprotein metabolism, cell differentiation, atherosclerosis, and apoptosis.(26–29) We have demonstrated previously that specific oxysterols stimulate the osteogenic differentiation of pluripotent MSCs and inhibit their adipogenic differentiation through the activation of Hedgehog signaling in vitro(30–33) and enhance bone healing in rat critical-sized calvarial defects in vivo.(34) Here, we report that osteogenic oxysterols are novel activators of expression of the Notch target genes *HES-1*, *HEY-1*, and *HEY-2* in MSCs. Moreover, the induction of Notch target gene expression by 20*S* is not mediated by the canonical Notch signaling pathway but mainly by Hedgehog signaling and in part by LXR signaling, and HES-1 and HEY-1 induction appears necessary for maximal induction of osteogenesis by 20*S*.

## Materials and Methods

### Cell culture and reagents

M2-10B4 (M2) pluripotent mouse marrow stromal cells and *Smo*^−/−^ mouse embryonic fibroblasts (MEFs) were maintained as described previously.(31,32,35,36) Cell treatment was performed in differentiation medium containing 5% fetal bovine serum (FBS), 50 µg/mL ascorbate, and 3 mM β-glycerophosphate. Oxysterols were purchased from Sigma-Aldrich, Co. (St. Louis, MO, USA); *N*-[*N*-(3,5-difluorophenacetyl-l-alanyl)] *S*-phenylglycine *t*-butyl ester (DAPT) and cyclopamine were from Calbiochem (La Jolla, CA, USA), and recombinant mouse Shh N-terminal peptide and Jagged-1 were from R&D Systems (Minneapolis, MN, USA).

### Quantitative reverse-transcriptase polymerase chain reaction (qRT-PCR)

Total RNA was extracted with an RNA isolation kit from Stratagene (La Jolla, CA, USA) according to the manufacturer's instructions. RNA was DNase treated using a DNA-free kit from Ambion (Austin, TX, USA). Then 3 µg of RNA was reverse-transcribed using reverse transcriptase from Stratagene (La Jolla, CA, USA) to make single-stranded cDNA. The cDNAs then were mixed with Qi SYBR Green Supermix (Bio-Rad, Hercules, CA, USA) for qRT-PCR assay using a Bio-Rad I-cycler IQ quantitative thermocycler. All PCR samples were prepared in triplicate wells in a 96 well plate. After 40 cycles of PCR, melt curves were examined to ensure primer specificity. Fold changes in gene expression were calculated using the ΔΔ*C*_*t*_ method and normalized to the expression of the housekeeping gene *GAPDH*. Primers used were as follows: *HES-1*: 5′-TACCCCAGCCAGTGTCAACA-3′ and 5′-CCATGATAGGCTTTGATGACTTTCT-3′(37); *HEY-1*: 5′-TGAGCTGAGAAGGCTGGTAC-3′ and 5′-ACCCCAAACTCCGATAGTCC-5′(38); *HEY-2*: 5V′-TGAGAAGACTAGTGCCAACAGC-3′ and 5′-TGGGCATCAAAGTAGCCTTTA-3′(38); *Jagged-1*: 5′-TGGTTGGCTGGGAAATTGA-3′ and 5′-TGGACACCAGGGCACATTC-3′(39); *Delta-1*: 5′-CACTATGGACAGTTGCTTTGAAGAGT-3′ and 5′-TGGCTCATAGTAATCCAAGATAGACG-5′(40); *Notch-1*: 5′-GGATCACATGGACCGATTGC-3′ and 5′-ATCCAAAAGCCGCACGATAT-3′(39); *Notch-2*: 5′-CCCCTTGCCCTCTATGTACCA-3′ and 5′-GGTAGGTGGGAAAGCCACACT-3′(39); *ALP*: 5′-AAACCCAGAACACAAGCATTCC-3′ and 5′-TCCACCAGCAAGAAGAAGCC-3′; *ABCA1*: 5′-TGCCACTTTCCGAATAAAGC-3′ and 5′-GGAGTTGGATAACGGAAGCA-3′; *BSP* 5′-ACGCCACACTTTCCACACTCTC-3′ and 5′-TTCCTCTTCCTCTTCTTCTTCTTCTTCC-3′; and *GAPDH*: 5′- ATGGACTGTGGTCATGAGCC-3′ and 5′- ATTGTCAGCAATGCATCCTG-3′.

### CBF-1 luciferase assay

M2 cells at 70% confluency in 24 well plates were transiently transfected with CBF-1 luciferase reporter construct pTK-luciferase plasmid and pTK-Renilla-luciferase plasmid (Promega, Madison, WI, USA) using Fugene 6 Transfection Reagents from Roche (Indianapolis, IN, USA).(2) Twenty-four hours after transfection, the cells were treated with control vehicle or Notch interacellular domain (NICD) overexpression vector with or without 5 µM 20*S* and 200 ng/mL mouse recombinant Shh for 24 and 48 hours, and Notch activation of CBF-1 was normalized to Renilla luciferase activity. Transfection efficiency was monitored by cotransfecting with a plasmid expressing green fluorescent protein.

### Jagged-1, Notch intracellular domain (NICD), and HES-1 Western blot

For Jagged-1 Western blot, M2 cells at confluence were treated with control vehicle (control), 5 µM 20(*S*)-hydroxycholesterol (20*S*), and 200 ng/mL sonic hedgehog (Shh). After 48 or 72 hours of treatment, whole-cell lysates were collected, and protein concentrations were determined using the Bio-Rad protein assay. For NICD Western blot, M2 cells at 100% confluence were treated with control vehicle (control) or 5 µM 20*S* or cultured on 5 µg/mL immobilized Jagged-1. After 48 and 72 hours, nuclear extracts were collected and protein concentrations determined using the Bio-Rad protein assay. For Western blotting of HES-1 and β-actin, whole-cell lysates were collected after 72 hours of control vehicle or 5 µM 20*S* treatment in M2 cells transfected with either scramble control or *HES-1* siRNA. The samples were subjected to sodium dodecylsulfate–polyacrylamide gel electrophoresis (SDS-PAGE) and transferred overnight onto a nitrocellulose membrane (Amersham Biosciences, Piscataway, NJ, USA). Blots then were incubated with polyclonal antibodies against HES-1 from Santa Cruz Biotechnology (Santa Cruz, CA, USA), and Jagged-1, NICD, and β-actin from Cell Signaling Technology (Danvers, MA, USA). As a positive control for the canonical Notch pathway activation, cells were cultured on immobilized Jagged-1 from R&D Systems to induce nuclear NICD accumulation.(40,41)

### Alkaline phosphatase activity assay

M2 cells at confluence were treated with control vehicle (control) or 5 µM 20*S*. For experiments with immobilized Jagged-1, M2 cells were cultured in tissue culture wells coated with 2.5 or 5 µg/mL Jagged-1.(40,41) After 72 hours, colorimetric alkaline phosphatase (ALP) activity assay on whole-cell extracts was performed as described previously.(30)

### LXR-α, LXR-β, HES-1, and HEY-1 siRNA transfection

Both *LXR-α* and *LXR-β* siRNAs (ON-TARGETplus SMARTpool Catalog No. L-040649-01-0010 and L-042839-00-0010) were obtained from Dharmacon (Lafayette, CO, USA). To knock down LXRs, M2 cells at 70% confluence in 6 well plates were transfected with siRNA using DharmaFECT transfection reagent (Dharmacon) to a final concentration of 25 nM of each siRNA.(42) Knockdown of target genes was monitored at the mRNA level by quantitative real-time PCR. At 100% confluence, transfected cells were treated with control vehicle or 5 µM 20*S*. After a 2 day incubation, *HES-1* and *HEY-1* mRNA expression was measured by quantitative real-time PCR. Both *HES-1* and *HEY-1* siRNAs were obtained from QIAGEN (Valencia, CA, USA). To knock down HES-1 or HEY-1, M2 cells at 70% confluence in 6 well plates were transfected with siRNA using DharmaFECT transfection reagent (Dharmacon) to a final concentration of 50 nM of each siRNA. At 100% confluence, transfected cells were treated with 5 µM 20*S*. After 3 days of incubation, alkaline phosphatase (*ALP*), bone sialoprotein (*BSP*), and osteocalcin (*OCN*) mRNA expression was measured by quantitative real-time PCR.

### Statistical analysis

Statistical analyses were performed using the StatView 5 program. All *p* values were calculated using ANOVA and Fisher's projected least-significant-difference (PLSD) significance test. A value of *p* < .05 was considered significant.

## Results

### 20(*S*)-hydroxycholesterol induces the expression of Notch target genes

In an initial microarray-based gene expression analysis using Affymetrix mouse 430A gene chips,(32) we found that treatment of M2 cells with an osteogenic oxysterol combination of 20*S* + 22(*S*)-hydroxycholesterol (5 µM each) for 48 hours induced the expression of the Notch target genes *HES-1* (*Hairy/Enhancer-of-Split 1*, NM 008235, 2.55-fold induction, *p* = .0017) and *HEY-2* (*Hairy/Enhancer-of-Split related with YRPW motif 2*, NM 013904, 2.6-fold induction, *p* = .0009). In recent studies, we have found that 20*S* is the most potent naturally occurring osteogenic oxysterol in our M2 cell system and that the osteogenic effects of 5 µM 20*S* used alone are quite significant, although less than those of 20*S* + 22*S* combination (data not shown). Hence further studies were performed with 20*S* alone.

**Fig. 1:**
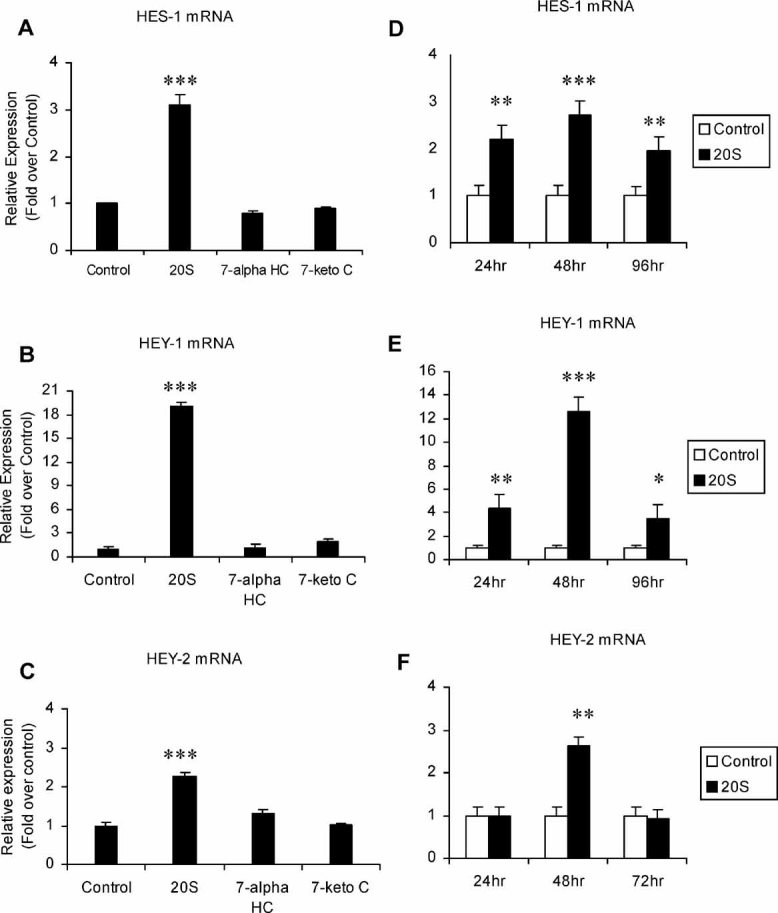
20(*S*)-Hydroxycholesterol (20*S*) induces Notch signaling target genes *HES-1*, *HEY-1*, and *HEY-2* in M2-10B4 bone marrow stromal cells. (*A–C*) M2 cells were treated at confluence with control vehicle or 5 µM 20*S*, 7α-hydroxycholesterol (7-αHC), or 7-ketocholesterol (7-ketoC) for 48 hours. *HES-1*, *HEY-1*, and *HEY-2* mRNA expression was measured by quantitative real-time PCR. (*D–F*) M2 cells were treated at confluence with control vehicle or 5 µM 20*S* for 24, 48, and 96 hours. *HES-1*, *HEY-1*, and *HEY-2* mRNA expression was measured by quantitative real-time PCR. Fold changes in gene expression compared with the control were calculated using the ΔΔ*C_t_* method and reported as the mean of triplicate determination ± SD (*A–C*: ***p < .0001 for control, 7α-HC or 7-keto C versus 20*S*; *D*: ***p < .0001 for control versus 20*S* at 48 hours; **p < .001 for control versus 20*S* at 24 and 96 hours. E: **p < .001 for control versus 20*S* at 24 hours; ***p < .0001 for control versus 20*S* at 48 hours; *p < .05 for control versus 20*S* at 96 hours. F: **p < .001 for control versus 20*S* at 48 hours).

To confirm the microarray data, we then examined the effect of 20*S* on Notch target gene expression in M2 cells using real-time PCR. 20*S* significantly induced *HES-1*, *HEY-1*, and *HEY-2* mRNA expression at 48 hours, whereas the nonosteogenic oxysterols 7α-hydroxycholesterol and 7-ketocholesterol did not induce these genes (Fig. 1A–C). Time-course studies showed that 20*S* significantly induced *HES-1* and *HEY-1* mRNA expression at 24, 48, and 96 hours in M2 cells, with maximum expression at 48 hours (see Fig. 1D, E), whereas significant induction of *HEY-2* mRNA expression was observed only at 48 hours (see Fig. 1F).

### Mechanism of HES-1 and HEY-1 induction by 20(*S*)-hydroxycholesterol

**Fig. 2:**
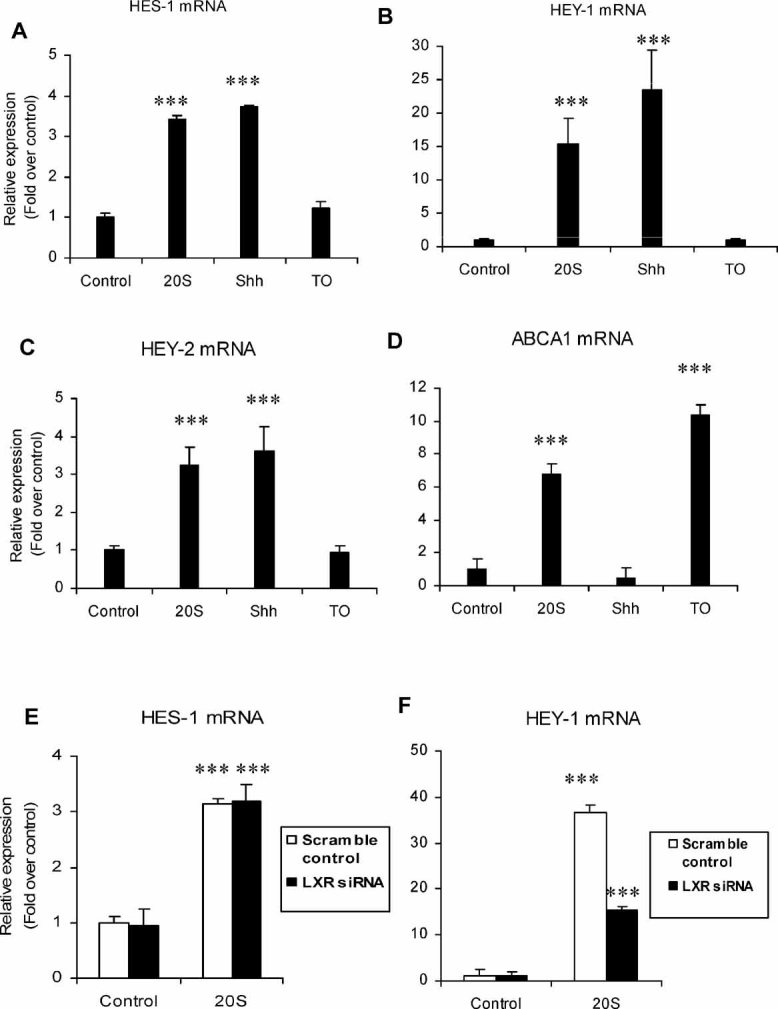
Mechanism of *HES-1* and *HEY-1* induction by 20(*S*)-hydroxycholesterol (20*S*). M2 cells were treated at confluence with control vehicle, 5 µM 20*S*, 200 ng/mL recombinant mouse sonic hedgehog (Shh), or 2 µM of LXR ligand TO901317 (TO) for 48 hours. *HES-1*, *HEY-1*, *HEY-2*, and *ABCA1* mRNA expression was measured by quantitative real-time PCR (*A–D*). For *LXR* siRNA experiments (*E*, *F*), M2 cells at 70% confluence were transfected with both *LXRα* and *LXRβ* siRNA to a final concentration of 25 nM of each siRNA. The scramble control or *LXR* siRNA–transfected cells were treated with control vehicle or 5 µM 20*S* for 48 hours. *HES-1* and *HEY-1* mRNA expression was measured by quantitative real-time PCR. Fold changes in gene expression compared with the control were calculated using the ΔΔ*C_t_* method and reported as the mean of triplicate determination ± SD. (*A–C*) ***p < .0001 for control versus 20*S* or Shh. (*D*) ***p < .0001 for control versus 20*S* or TO. (*E*) ***p < .0001 for control versus 20*S* with or without *LXR* siRNA. (*F*) ***p < .0001 for control versus 20*S* with and without *LXR* siRNA and for 20*S* in the presence of scrambled versus *LXR* siRNA.

**Fig. 3:**
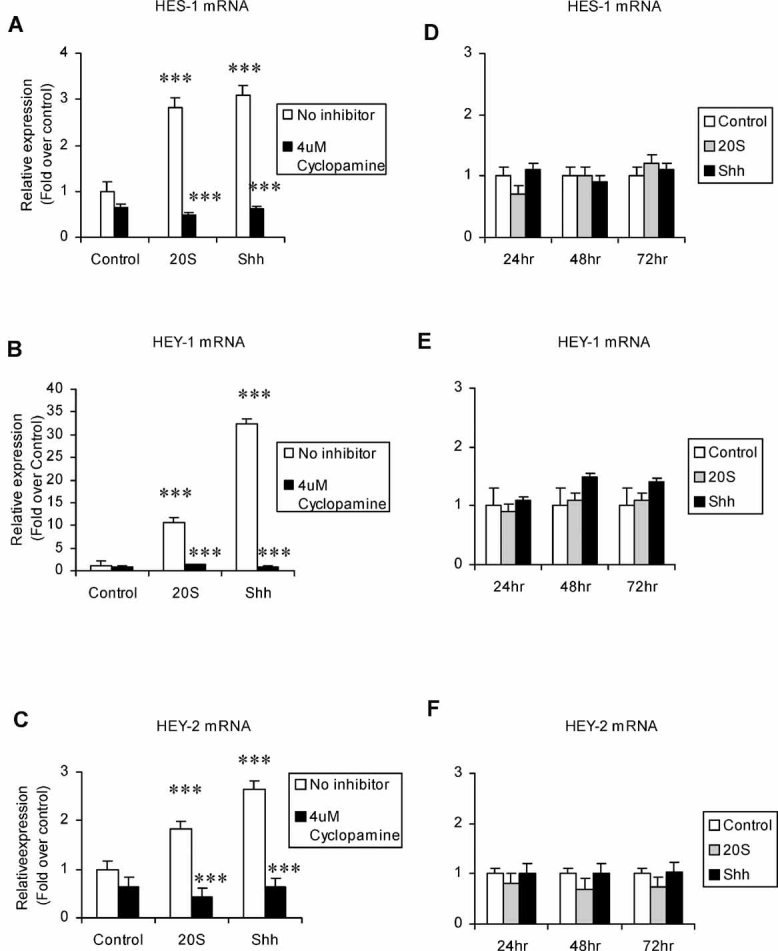
20(*S*)-Hydroxycholesterol (20*S*) induces Notch target gene expression through Hedgehog signaling. (*A–C*) M2 cells, which do express *Smoothened* (*Smo*^+/+^), were treated at confluence with control vehicle (control), 5 µM 20*S*, or 200 ng/mL recombinant mouse sonic hedgehog (Shh) with or without a 2 hour pretreatment with cyclopamine. After 48 hours of treatment, *HES-1*, *HEY-1*, and *HEY-2* mRNA expression was measured by quantitative real-time PCR. (*D–F*) *Smoothened* (−/−) mouse embryonic fibroblasts (*Smo*^−/−^ MEFs) at confluence were treated with control vehicle (control), 5µM 20S, or 200ng/mL Shh. After 24, 48, and 72 hours of treatment, *HES-1*, *HEY-1*, and *HEY-2* mRNA expression was measured by quantitative real-time PCR. Fold changes in gene expression relative to control cells were calculated using the ΔΔ*C_t_* method and reported as the mean of triplicate determination ± SD. (*A–C*) ***p < .0001 for control versus 20*S* or Shh and for 20*S* and Shh each in the presence versus absence of cyclopamine.

Since osteogenic oxysterols are novel activators of Hedgehog,(32) as well as liver X receptor (LXR) signaling,(44) and Shh induces Notch receptors and *HES-1* expression,(20–22) we examined whether the induction of *HES-1*, *HEY-1*, and *HEY-2* mRNA expression in MSCs occurs through the Hedgehog or LXR signaling pathway. 20*S* and Shh induced the expression of all three Notch target genes in M2 cells, whereas the synthetic LXR agonist TO-901317 (TO) did not induce the expression of these genes, suggesting that the induction of Notch target genes by 20*S* is mainly through Hedgehog signaling and not through LXR signaling (Fig. 2A–C). TO activation of LXR under these conditions was confirmed by a 10-fold increase in the mRNA expression of the LXR target gene *ABCA1* compared with control cells (see Fig. 2D). Since nuclear hormone receptor conformation may vary depending on the ligand used, and since the effect of 20*S* on LXR conformation and activity may differ from what is caused by TO,(43) we further examined the potential role of LXR in mediating oxysterol-induced Notch target gene expression in M2 cells using siRNA to knock down LXRα and LXRβ expression in these cells, as we have previously reported.(42) Results showed that 20*S*-induced *HES-1* expression was not affected by *LXR* siRNAs (see Fig. 2E), whereas *HEY-1* expression was significantly inhibited by *LXR* siRNA (see Fig. 2F), suggesting the role of LXR as well as Hedgehog signaling in 20*S*-induced *HEY-1* but not *HES-1* expression. To further confirm that 20*S* induces *HES-1*, *HEY-1*, and *HEY-2* mRNA expression mainly through the Hedgehog signaling pathway, M2 cells were treated with cyclopamine, a specific inhibitor of the Hedgehog signaling pathway that binds directly to and inhibits Smoothened.(32) Results showed that cyclopamine completely blocked 20*S* and Shh induction of *HES-1*, *HEY-1*, and *HEY-2* mRNA expression (Fig. 3A–C). We also examined whether 20*S* and Shh could induce the expression of Notch target genes in *Smo*^−/−^ mouse embryonic fibroblasts (*Smo*^−/−^ MEFs), in which activation of Hedgehog signaling cannot occur. 20*S* and Shh did not induce *HES-1*, *HEY-1*, and *HEY-2* mRNA expression at all time points tested in *Smo*^−/−^ MEFs (see Fig. 3D–F), suggesting that oxysterol induction of Notch target genes requires the activation of the Hedgehog signaling pathway.

### 20(*S*)-Hydroxycholesterol induces Notch target gene expression independent of the canonical Notch signaling pathway

**Fig. 4:**
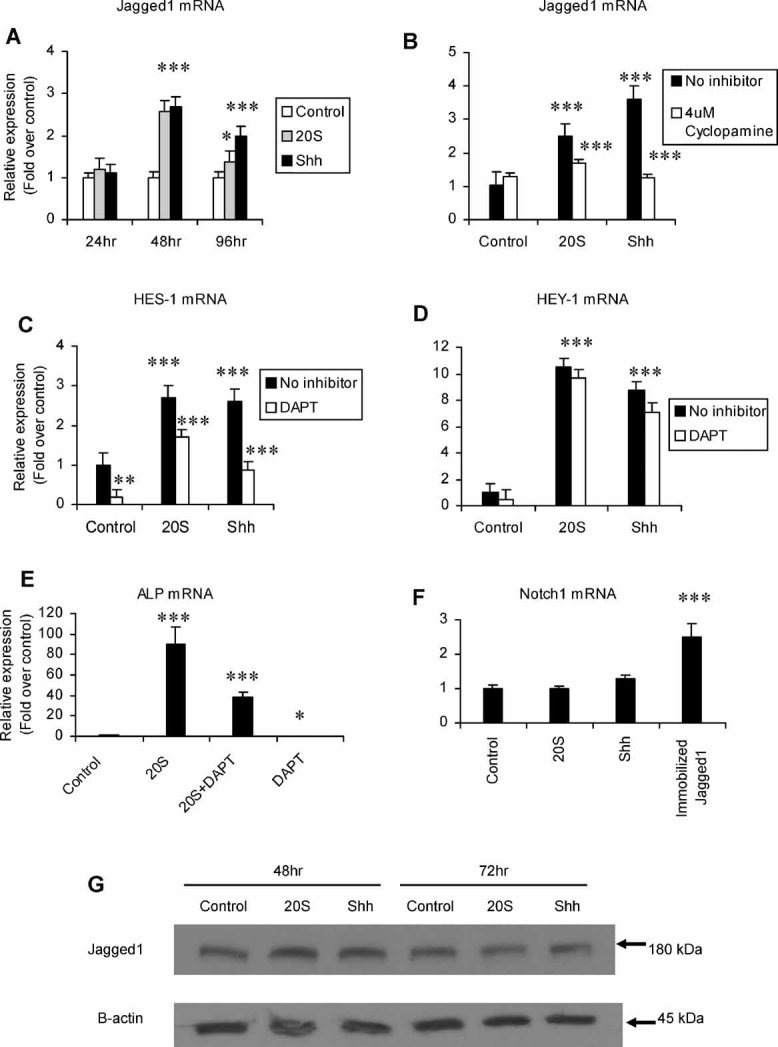
Effects of 20(*S*)-hydroxycholesterol (20*S*), sonic hedgehog (Shh), and DAPT on Jagged-1 and Notch target gene expression. (*A*) M2 cells at confluence were treated with control vehicle (control), 5 µM 20S, or 200 ng/mL Shh. After 24, 48, and 96 hours of treatment, *Jagged-1* mRNA expression was measured by quantitative real-time PCR. (*B*) M2 cells at confluence were treated with control vehicle (control), 5 µM 20*S*, or 200 ng/mL Shh with or without a 2 hour pretreatment with cyclopamine. After 48 hours of treatment, *Jagged-1* mRNA expression was measured by quantitative real-time PCR. (*C*, *D*) M2 cells at confluence were treated with control vehicle (control), 5 µM 20*S*, or 200 ng/mL (Shh) with or without a 2 hour pretreatment with 10 µM DAPT. After 48 hours of treatment, *HES-1* and *HEY-1* mRNA expression was measured by quantitative real-time PCR. Fold changes in gene expression relative to control cells were calculated using the ΔΔ*C_t_* method and reported as the mean of triplicate determination ± SD. (*E*) M2 cells at confluence were treated with control vehicle (control) or 5 µM 20*S* with or without a 2 hour pretreatment with 10 µM DAPT. After 6 days of treatment, alkaline phosphatase (*ALP*) mRNA expression was measured by quantitative real-time PCR. (*F*) M2 cells were treated with control vehicle (control), 5 µM 20*S*, or 200 ng/mL Shh. Immobilized Jagged-1 was used as a positive control. After 48 hours of treatments, *Notch-1* mRNA expression was measured by quantitative real-time PCR. (*G*) M2 cells at confluence were treated with control vehicle (control), 5 µM 20*S*, or 200 ng/ml Shh for 48 or 72 hours. Whole-cell lysates were collected for Western blotting using antibodies to Jagged-1 and β-actin. (*A*) ***p < .0001 for control versus 20*S* or Shh at 48 hours and for control versus Shh at 96 hours. *p < 0.05 control version 20*S* at 96 hours. (*B*) ***p < .0001 for control versus 20*S* or Shh and for 20*S* and Shh in the presence versus absence of cyclopamine. (*C*) ***p < .0001 for control versus 20*S* or Shh in the absence of DAPT and for 20*S* and Shh in the presence versus absence of DAPT; **p < .001 for control versus 20S versus Shh all in the presence of DAPT. (*D*) ***p < .0001 for control versus 20*S* and Shh in the presence or absence of DAPT. (*E*) ***p < .0001 for control and 20*S* + DAPT versus 20*S* and *p < .05 for control versus DAPT. (*F*) ***p < .0001 for control versus immobilized Jagged-1.

In order to determine if 20*S* and Shh induce Notch target gene expression in M2 cells through the canonical Notch signaling pathway, we examined whether 20*S* and Shh induce the mRNA expression of ligands and the Notch receptors Jagged-1 and -2; Delta-1, -3, and -4; and Notch-1, -2, -3, and -4 that interact to activate the canonical Notch signaling.(1,2) Although mRNA for all the Notch receptors (Notch-1, -2, -3, and -4) and ligands (Jagged-1 and -2 and Delta-1, -3, and -4) are present in M2 cells (data not shown), 20*S* and Shh caused only a significant induction of *Jagged-1* mRNA expression at 48 and 96 hours (Fig. 4A) but not *Notch-1* (Fig. 4F) or any of the other Notch signaling receptors or ligands (data not shown). In addition, induction of *Jagged-1* mRNA expression by 20*S* and Shh was completely blocked by cyclopamine (see Fig. 4B), indicating that 20*S* and Shh both induce Jagged-1 expression through Hedgehog signaling–dependent mechanisms. However, Jagged-1 protein expression was not increased by 20*S* or Shh compared with the control cells at 48 and 72 hours (see Fig. 4G).

To further investigate the potential involvement of canonical Notch signaling in the osteogenic response of MSCs to 20*S* and Shh, we examined the effect of *N*-[*N*-(3,5-difluorophenacetyl-l-alanyl)] *S*-phenylglycine *t*-butyl ester (DAPT), a canonical Notch signaling inhibitor, on 20*S* and Shh induction of Notch target gene expression to determine whether this process requires the activation of Notch receptors and the production of Notch intracellular domain (NICD). DAPT blocks canonical Notch signaling by inhibiting γ-secretase activity and the production of NICD when Notch ligands bind to Notch receptor.(45) DAPT treatment significantly reduced both 20*S* and Shh induction of *HES-1* mRNA expression, as well as baseline *HES-1* mRNA expression compared with control cells (see Fig. 4C). However, 20*S* and Shh still significantly induced *HES-1* expression in the presence of DAPT (DAPT alone versus 20*S* + DAPT or Shh + DAPT). In contrast to its effects on *HES-1* expression, DAPT did not inhibit baseline or 20*S*- and Shh-induced levels of *HEY-1* mRNA expression (see Fig. 4D), suggesting that 20*S* and Shh induction of *HEY-1* mRNA expression does not require the canonical Notch signaling pathway. It must be noted that *HEY-1* mRNA is expressed at a much lower level than *HES-1* in control M2 cells (data not shown) and hence the lack of DAPT effect on its low baseline expression. In addition, we found that DAPT significantly inhibited 20*S*-induced *ALP* mRNA expression on day 6 (see Fig. 4E), suggesting that baseline Notch signaling is important for 20*S* to fully induce osteogenic differentiation in M2 cells.

**Fig. 5:**
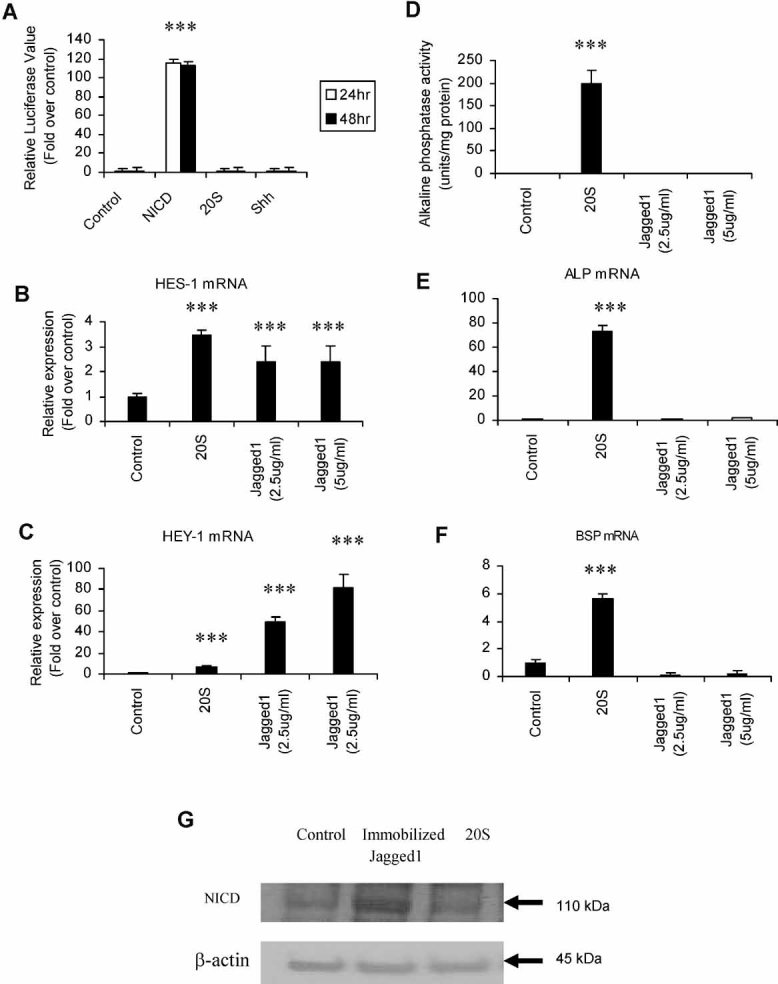
20(*S*)-Hydroxycholesterol (20*S*) and Shh induce Notch target genes independent of the canonical Notch signaling pathway. (*A*) M2 cells at 70% confluence in a 24 well plate were transiently transfected with CBF-1 luciferase reporter construct pTK-luciferase plasmid and pTK-Renilla-luciferase plasmid (Promega, Madison, WI, USA) using Fugene 6 Transfection Reagents from Roche (Indianapolis, IN, USA). Twenty-four hours after transfection, the cells were treated with control vehicle, Notch interacellular domain (NICD) overexpression vector, 5 µM 20*S*, or 200 ng/mL Shh for 24 and 48 hours. Notch activation of CBF-1 was normalized to Renilla luciferase activity. Transfection efficiency was monitored by cotransfecting with a plasmid expressing green fluorescent protein. (*B*, *C*, *E*, *F*) M2 cells were treated at confluence with control vehicle or 5 µM 20*S* or cultured on 2.5 or 5 µg/mL immobilized Jagged-1 for real-time PCR and alkaline phosphatase (ALP) activity analyses. After 48 hours of treatment, *HES-1*, *HEY-1*, *ALP*, and bone sialoprotein (*BSP*) mRNA expression was measured by quantitative real-time PCR. (*D*) After 72 hours of treatment, *ALP* activity using whole-cell extracts was measured by a colorimetric method. Fold changes in gene expression compared with the control cells were calculated using the ΔΔ*C_t_* method and reported as the mean of triplicate determination ± SD. (*G*) M2 cells at confluence were treated with control vehicle (control), cultured on 5 µg/mL immobilized Jagged-1, or treated with 5 µM 20*S*. After 48 and 72 hours of treatment, nuclear extracts were collected for Western blotting using antibodies to NICD and β-actin. (*A*) ***p < .0001 for control versus NICD. (*B*) ***p < .0001 for control versus 20*S* and Jagged-1 (2.5 and 5 µg/mL). (*C*) **p < .001 for control versus 20*S*; ***p < .0001 for control versus Jagged-1 (2.5 and 5 µg/mL). (*D–F*) ***p < 0.0001 for control versus 20*S*.

To further confirm the absence of canonical Notch signaling in M2 cell responses to 20*S* and Shh, we examined the effects of 20*S* and Shh on CBF-1 luciferase reporter activity, nuclear localization of NICD, and the expression of Notch target genes and osteogenic genes (Fig. 5). If 20*S* or Shh induction of Notch target gene expression is mediated at least in part through canonical Notch signaling, it would be expected that CBF-1 luciferase activity would be induced by 20*S* and Shh and that NICD protein accumulation would be increased in nuclear extracts from 20*S*- and Shh-treated M2 cells. NICD overexpression was used as a positive control for the activation of CBF-1 luciferase reporter activity, and immobilized Jagged-1 was used as a positive control to stimulate canonical Notch signaling and nuclear NICD accumulation. We found that neither 20*S* nor Shh induced CBF-1 luciferase reporter activity, whereas NICD overexpression caused a robust increase in reporter activity (see Fig. 5A). Moreover, 20*S* did not cause an increase in nuclear levels of NICD protein, whereas cells cultured on immobilized Jagged-1 exhibited increased NICD accumulation in the nucleus (see Fig. 5G), and DAPT treatment inhibited baseline NICD production and accumulation in the nucleus, as expected (data not shown).

Finally, we examined the induction of the expression of Notch target genes in parallel with osteogenic genes by 20*S* and immobilized Jagged-1. Both 20*S* and immobilized Jagged-1 significantly increased *HES-1* and *HEY-1* mRNA expression (see Fig. 5B, C). However, whereas 20*S* induced the mRNA expression of the osteogenic genes alkaline phosphatase (*ALP*) and bone sialoprotein (*BSP*), immobilized Jagged-1 did not affect osteogenic gene expression (see Fig. 5D–F), indicating that canonical Notch signaling per se does not stimulate osteogenic differentiation of MSCs. In addition, we found that culturing M2 cells on immobilized Jagged-1 had no effects on mineralization at baseline or when induced by 20*S* (data not shown).

### HES-1 and HEY-1 knockdown significantly inhibits 20(*S*)-hydroxycholesterol-induced osteogenesis in MSCs

**Fig. 6:**
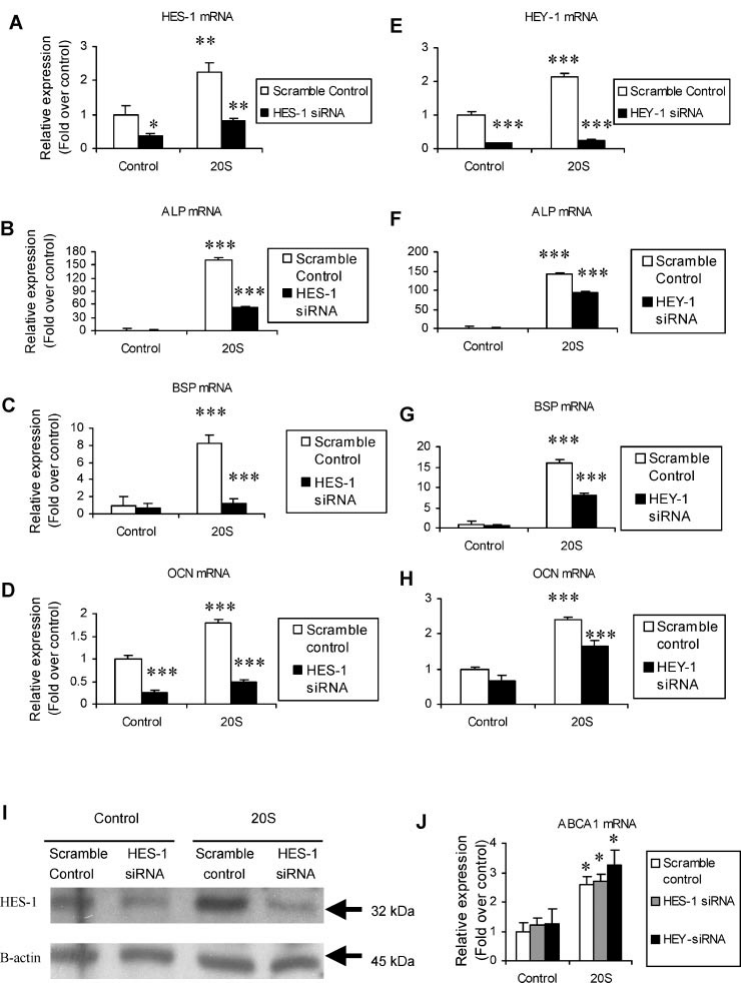
*HES-1* and *HEY-1* siRNA significantly inhibit 20(*S*)-hydroxycholesterol (20*S*)–induced osteogenic gene expression in MSCs. To knock down *HES-1* and *HEY-1* expression, M2 cells at 70% confluence were transfected with siRNA to a final concentration of 50 nM of either siRNA. At 100% confluence, transfected cells were treated with control vehicle or 5 µM 20*S* for 3 days. After 3 days of incubation, *HES-1*, *HEY-1* (*A*, *E*), alkaline phosphatase (*ALP*) (*B*, *F*), bone sialoprotein (*BSP*) (*C*, *G*), and *ABCA1* mRNA expression (*I*) was measured by quantitative real-time PCR. Fold changes in gene expression relative to control cells were calculated using the ΔΔ*C_t_* method and reported as the mean of triplicate determination ± SD. Osteocalcin (*OCN*) mRNA expression was measured after 6 days of incubation (*D*, *H*). For Western blotting of HES-1 and β-actin, whole-cell lysates were collected after 72 hours of control vehicle or 5 µM 20*S* treatment in M2 cells transfected with either scramble control or *HES-1* siRNA (*J*). (*A*) **p < .001 for control versus 20*S* and for 20*S* in the presence of scrambled versus *HES-1* siRNA; *p < .05 for control in the presence of scrambled versus *HES-1* siRNA. (*B*, *C*) ***p < .0001 for control versus 20*S* in the presence of scrambled or *HES-1* siRNA and for 20*S* in the presence of scrambled versus *HES-1* siRNA. (*D*) ***p < .0001 for control in the presence of scrambled versus *HES-1* siRNA and for 20*S* in the presence of scrambled versus *HES-1* siRNA and for control versus 20*S* in the presence of scrambled siRNA. (*E*) ***p < .0001 for control in the presence of scrambled versus *HEY-1* siRNA and for 20*S* in the presence of scrambled versus *HEY-1* siRNA and for control versus 20*S* in the presence of scrambled siRNA. (*F*, *G*) ***p < .0001 for control versus 20*S* in the presence and absence of *HEY-1* siRNA; ***p < .001 for 20*S* in the presence of scrambled versus *HEY-1* siRNA. (*H*) ***p < .0001 for control versus 20*S* in the presence of scrambled siRNA and for 20*S* in the presence of scrambled versus *HEY-1* siRNA. (*J*) *p < .05 for control versus 20*S* in the presence of scrambled or either *HES-1* or *HEY1* siRNA.

We used siRNA gene knockdown studies to determine whether increased *HES-1* and/or *HEY-1* expression is required for 20*S* induction of osteogenic differentiation in M2 cells. Treatment with *HES-1* or *HEY-1* siRNA was found to reduce 20*S* induction of *HES-1* and *HEY-1* mRNA expression, respectively, by over 70% at 3 days compared with control scrambled siRNA-transfected cells (Fig. 6A, E). Western blot analysis of HES-1 protein expression also showed that *HES-1* siRNA reduced baseline as well as 20*S*-induced HES-1 protein levels (see Fig. 6I). Moreover, *HES-1* and *HEY-1* siRNA did not inhibit 20*S*-induced *ABCA1* mRNA expression (see Fig. 6J), indicating that siRNA knockdown was specific to *HES-1* and *HEY-1* and not due to any toxic effects. *HES-1* siRNA treatment inhibited 20*S*-induced mRNA expression of the osteogenic genes *ALP*, *BSP*, and *OCN* by 70%, 90%, and 73%, respectively (see Fig. 6B–D), although baseline expression of *OCN* also was inhibited to the same degree. In addition, treatment with *HEY-1* siRNA also caused a significant inhibition of *ALP*, *BSP*, and *OCN* mRNA expression, although to a lesser extent than that achieved by *HES-1* siRNA (see Fig. 6F–H), suggesting that both *HES-1* and *HEY-1* play a significant role in 20*S* induction of osteogenesis in MSCs.

## Discussion

In this study we demonstrated that 20*S* induces the expression of the Notch target genes *HES-1*, *HEY-1*, and *HEY-2* in murine M2 MSCs. Induction of Notch target gene expression by 20*S* appears to be mediated through Hedgehog signaling because cyclopamine, a Hedgehog pathway inhibitor, completely blocked 20*S* induction of Notch target gene expression, and 20*S* did not induce Notch target genes in *Smo*^−/−^ MEFs, which cannot generate Hedgehog pathway signaling. However, unlike *HES-1*, *LXR* activation by 20*S* appears to cooperate with Hedgehog signaling to induce *HEY-1* expression in response to 20*S* and therefore is inhibited significantly by *LXR* siRNA. A similar pattern of responses was observed when MSCs were treated with Shh. A recent study in C3H10T1/2 embryonic fibroblasts and MNS70 neuronal cells by Ingram and colleagues(22) suggested that Shh regulates *HES-1* expression through a mechanism that is independent of canonical Notch signaling.(22) It was shown that DAPT decreased baseline HES-1 levels in both cell types but that there was no reduction in the fold change in *HES-1* mRNA expression induced by Shh treatment in the presence of DAPT, suggesting that DAPT inhibited baseline and not Shh-induced *HES-1* expression.(22) Moreover, it was reported recently that Shh directly regulates *HES-1* expression in retinal progenitor cells through a Gli2-dependant and Notch-independent mechanism.(24) Based on similar findings in our present studies, we also conclude that in MSCs, the canonical Notch signaling inhibitor DAPT inhibits baseline but not 20*S*- or Shh-induced *HES-1* expression. Moreover, we also found that DAPT treatment significantly inhibited baseline as well as 20*S*-induced *ALP* mRNA expression on day 6, which most likely is due to the inhibition of baseline *HES-1* expression with minimal, if any, effects on 20*S*-induced *HES-1* expression.

In addition, DAPT did not inhibit 20*S*- or Shh-induced *HEY-1* expression, whereas cyclopamine completely inhibited its expression. The lower baseline level of expression of *HEY-1* compared with *HES-1* and the inability of DAPT to inhibit baseline *HEY-1* expression while inhibiting baseline *HES-1* expression suggest differences in transcriptional regulation of these Notch target genes in MSCs. Our data also showed that 20*S* and Shh induction of Notch target genes was not accompanied by an increase in CBF-1 luciferase reporter activity or NICD protein accumulation in the nucleus, indicating that the induction of Notch target gene expression by 20*S* or Shh occurs independent of NICD-CBF-1 activation. Although we found that 20*S* did moderately induce *Jagged-1* mRNA expression, Jagged-1 protein levels were not induced by 20*S* or Shh beyond baseline levels. This finding confirms the lack of any increase in either CBF-1 reporter activity or nuclear content of NICD, which would have been caused if Jagged-1 protein expression was induced by 20*S* or Shh. In a preliminary screen of 5 kb upstream of the transcription start site in mouse *HES-1* and *HEY-1* genes, we found several potential Gli-binding sites (data not shown), suggesting that Gli may directly regulate the expression of Notch target genes. Indeed, a recent report demonstrated direct binding of Gli2 to *HES-1* promoter in retinal progenitor cells treated with Shh.(24) Altogether, our findings demonstrate and confirm the activation of Notch target genes through a Hedgehog signaling–dependent mechanism in MSCs.

Previously we reported that specific oxysterols stimulate osteogenic differentiation of MSCs through various signaling pathways, including Hedgehog, Wnt, PKC, PKA, and PI3K.(32,33,40) The present study suggests that induction of *HES-1* and *HEY-1* expression by the osteogenic oxysterol 20*S* mediates stimulation of osteogenic differentiation of MSCs because *HES-1* and *HEY-1* knockdown significantly reduced oxysterol induction of the expression of the osteogenic genes *ALP*, *BSP*, and *OCN*. A potential role for *HES-1* in the regulation of the osteogenic differentiation of pluripotent MSCs has been reported previously.(5,15,46) McLarren and colleagues(15) demonstrated that *HES-1* physically interacts with *RUNX2*, a master regulator of osteogenesis, and potentiates *RUNX2*-mediated transcriptional activity by interfering with interaction of *RUNX2* with corepressors (TLE proteins).(15) Moreover, stimulation of *HES-1* expression by canonical Notch signaling has been shown to increase BMP-2-induced ALP activity and type I collagen and *RUNX2* mRNA expression, whereas inhibition of Notch signaling by the dominant-negative extracellular domain of *Notch-1* or *HES-1* siRNA significantly reduced BMP-2-induced responses.(5) These reports are consistent with a positive regulatory role for *HES-1* in oxysterol-induced osteogenesis. Although it is plausible that 20*S*-induced *HES-1* expression enhances osteogenic differentiation of MSCs through positive regulation of Runx2, future studies will examine this possibility directly.

*HEY-1* also may play a regulatory role in osteogenic differentiation. Our present studies suggest that the induction of *HEY-1* by 20*S* oxysterol plays an important role in 20*S*-induced osteogenic differentiation of MSCs. This finding is in agreement with the results of a recent study of BMP-9 induction of osteogenic differentiation in pluripotent C3H10T1/2 embryonic fibroblasts.(17) Sharff and colleagues demonstrated that BMP-9 significantly induced *HEY-1* at an early stage of BMP-9 induction of osteogenic differentiation. *HEY-1* knockdown caused the inhibition of BMP-9-induced osteogenic differentiation both in vitro and in vivo, whereas *HEY-1* overexpression increased BMP-9-mediated stimulation of late-stage mineralization of bone matrix.(17) It also was demonstrated that *HEY-1* and *RUNX2* synergistically increased BMP-9-induced osteogenic differentiation.(17) However, other studies have suggested that *HEY-1* may act as a negative regulator of osteogenic differentiation.(18,47) For example, it has been shown that BMP-2 stimulates *HEY-1* expression in mouse MC3T3 and C2C12 cells and that *HEY-1* gene knockdown with siRNA significantly increases bone matrix mineralization, suggesting that *HEY-1* is a negative regulator of osteoblast maturation.(47)

In vivo studies of the role of Notch signaling in bone formation also have resulted in complex findings. Recently, Hilton and colleagues(18) showed that mice lacking the key Notch signaling components Presenilin-1 and -2 and Notch-1 and -2 have excessive cancellous bone development and a decreased number of bone marrow mesenchymal progenitors compared with WT mice, suggesting that Notch signaling may play a positive role in maintaining the mesenchymal cell progenitor cell population while inhibiting its osteogenic maturation.(18) However, the decreased progenitor pool observed in Notch-deficient mice is associated with severe bone loss with age, suggesting that Notch signaling is important in the long-term maintenance of a pool of mesenchymal cell progenitors required for bone homeostasis.(18) Thus, although short-term inhibition of Notch signaling might augment bone formation by enhancing osteoblast maturation, in the longer term, Notch signaling appears to be essential for coordinating maintenance of the mesenchymal cell progenitor pool and the proper regulation of osteoblastogenesis and bone formation. Moreover, it has been reported that mice deficient in Dll-3 or Presenilin-1, key components of the canonical Notch signaling pathway, have severely impaired skeletal development, suggesting that Notch signaling is important in this process.(8,13,14) Accordingly, although the Notch signaling pathway appears to play an important role in osteogenic differentiation and bone formation, the specific mechanisms of its actions in this regard remain undefined. Furthermore, since we found that activation of canonical Notch signaling did not induce osteogenic differentiation of MSCs in this study, it will be important to further identify the interactions between canonical Notch signaling and other signal-transduction pathways that result in enhanced osteogenesis and bone formation. Our present findings suggest that canonical Notch signaling may act in cooperation with Hedgehog signaling to positively regulate osteogenesis.

**Fig. 7:**
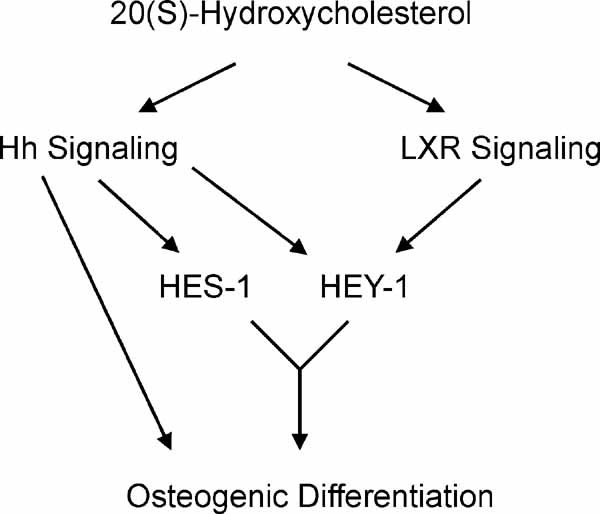
Regulation of osteogenic differentiation of bone marrow stromal cells (MSCs) by 20(*S*)-hydroxycholesterol (20*S*). Osteogenic oxysterol 20*S* induces Notch target gene expression in MSCs mainly through activation of hedgehog (Hh) signaling and in part through LXR signaling. 20*S*-induced osteogenesis is regulated in part by Notch target genes *HES-1* and *HEY-1*.

In summary, our studies demonstrated that the osteogenic oxysterol 20*S* and Shh both induce the expression of the Notch target genes *HES-1*, *HEY-1*, and *HEY-2* in MSCs both through activation of Hedgehog signaling and via a pathway independent of canonical Notch signaling (Fig. 7). Interestingly, as suggested by our studies using siRNA knockdown of LXRs, we found that *LXR* activation by 20*S* plays a role in the induction of *HEY-1* but not *HES-1* mRNA expression. Such effects of *LXR* activation on *HEY-1* expression is perhaps through cooperation with 20*S*-induced Hedgehog signaling because induction of *HEY-1* expression in cells cultured on immobilized Jagged-1, which does not stimulate Hedgehog pathway activity, did not induce osteogenesis. In addition, our studies showed that *HES-1* and *HEY-1* play a significant role in oxysterol-induced osteogenic differentiation of MSCs. Given the differences in the reports of the role of Notch target genes in osteogenesis, various experimental systems, and their apparent role in mediating the osteogenic effects of oxysterols in vitro, future mechanistic in vivo studies are required to determine the role of *HES* and *HEY* genes in oxysterol-induced osteogenesis and bone formation. Furthermore, given the subtle but important differences in osteogenic programs used by osteoblasts derived from the neural crest (e.g., calvarial osteoblasts) versus bone marrow mesenchymal cells, it will be important that future studies that elucidate the in vivo molecular mechanisms of oxysterol-induced osteogenesis also determine any differences that might exist in oxysterol actions when targeting osteoprogenitors from different origins. Development of an improved understanding of the molecular mechanisms by which osteogenic oxysterols stimulate the osteogenic differentiation of MSCs should enhance current understanding of the regulation of osteogenesis and potentially could lead to the development of novel oxysterol-based therapies for interventions in osteoporosis and enhancement of bone healing.
